# Infection Dynamics of *Clostridium perfringens* Fingerprinting in Buffalo and Cattle of Punjab Province, Pakistan

**DOI:** 10.3389/fvets.2022.762449

**Published:** 2022-07-22

**Authors:** Muhammad Umar Zafar Khan, Shumaila Khalid, Muhammad Humza, Shunli Yang, Mughees Aizaz Alvi, Tahir Munir, Waqar Ahmad, Muhammad Zahid Iqbal, Muhammad Farooq Tahir, Yongsheng Liu, Jie Zhang

**Affiliations:** ^1^Hebei Key Laboratory of Preventive Veterinary Medicine, College of Animal Science and Technology, Hebei Normal University of Science and Technology, Qinhuangdao, China; ^2^Institute of Microbiology, University of Agriculture, Faisalabad, Pakistan; ^3^Livestock and Dairy Development Department, Lahore, Pakistan; ^4^Key Laboratory of Agro-Products Quality and Safety Control in Storage and Transport Process, Ministry of Agriculture and Rural Affairs, Institute of Food Science and Technology, Chinese Academy of Agricultural Sciences, Beijing, China; ^5^Department of Plant Pathology, University of Agriculture, Faisalabad, Pakistan; ^6^State Key Laboratory of Veterinary Etiological Biology of Veterinary Parasitology of Gansu Province, Lanzhou Veterinary Research Institute, Chinese Academy of Agricultural Sciences, Lanzhou, China; ^7^Jiangsu Co-Innovation Center for the Prevention and Control of Important Animal Infectious Disease and Zoonoses, Yangzhou University, Yangzhou, China; ^8^Department of Clinical Medicine and Surgery, University of Agriculture, Faisalabad, Pakistan; ^9^The Equine Clinic, Al-Hashar Stables, Muscat, Oman; ^10^Department of Veterinary Medicine, University of Veterinary and Animal Sciences, Lahore, Pakistan; ^11^Health Security Partners, Washington, DC, United States

**Keywords:** *Clostridium perfringens*, sampling zones, intestinal contents, toxinotyping, PFGE

## Abstract

*Clostridium perfringens* produces core virulence factors that are responsible for causing hemorrhagic abomasitis and enterotoxemia making food, animals, and humans susceptible to its infection. In this study, *C. perfringens* was isolated from necropsied intestinal content of buffalo and cattle belonging to four major bovine-producing regions in the Punjab Province of Pakistan for the purpose offind out the genetic variation. Out of total 160 bovine samples (*n*: 160), thirty-three (*n*: 33) isolates of *C. perfringens* were obtained from buffalo (*Bubales bubalis*) and cattle (*Bos indicus*) that were further subjected to biochemical tests; 16S rRNA based identification and toxinotyping was done using PCR (Polymerase Chain Reaction) and PFGE (Pulse Field Gel Electrophoresis) pulsotypesfor genetic diversity. Occurrence of *C. perfringens* was found to be maximum in zone-IV (Bhakkar and Dera Ghazi Khan) according to the heatmap. Correlation was found to be significant and positive among the toxinotypes (α*-toxin*, and ε*-toxin*). Response surface methodology (RSM) *via* central composite design (CCD) and Box-Behnken design (BBD) demonstrated substantial frequency of *C. perfringens* based toxinotypes in all sampling zones. PFGE distinguished all isolates into 26 different pulsotypes using *SmaI* subtyping. Co-clustering analysis based on PFGE further decoded a diversegenetic relationship among the collected isolates. This study could help us to advance toward disease array of *C. perfringens* and its probable transmission and control. This study demonstrates PFGE patterns from Pakistan, and typing of *C. perfringens* by PFGE helps illustrate and mitigate the incidence of running pulsotypes.

## Introduction

Pakistan is an agriculture-based country and its livestock industry occupies a pivotal place in the economy, contributing 11.7% in the total gross domestic product (GDP) during the financial year 2019–2020 (1). In Pakistan, there are currently 90.8 million heads of buffalo and cattle, sharing 96.87 and 48.91% of the total milk and beef gross production, respectively (2). Due to the heavy potential of the livestock sector, a major proportion of the Pakistani population is directly or indirectly associated with the livestock sector. *C. perfringens* is an important etiological agent of causing different diseases in animals and humans (3). Diarrhea and pneumonia are considered to be a primary cause of mortality and morbidity in dairy calf worldwide (4). In domestic species, *C. perfringens* is the main cause of enteritis and enterotoxemia (5). In developed countries, food poisoning by *C. perfringens type A* is frequently reported foodborne illness; however, this type of food poisoning is not much reported in the developing countries (6, 7).

In recent times, the nomenclature of *C. perfringens* was augmented to seven types (A–G) by the addition of *C. perfringens* enterotoxin (*CPE*) and *NetB* hauling strains as peculiar toxin types (8). The pathogenic clostridial species can be classified into three groups, based on their toxin activity (enterotoxic, histotoxic, neurotoxic) on target tissues (9). *C. perfringens type A* has been and is still frequently blamed for enteritis, abomasitis, and/or enterotoxemia in cattle (10). The coding gene (*CPA*) is highly conserved and thus present in all strains of *C. perfringens* (11, 12). *CPA* plays a critical role in the pathogenesis of gas gangrene in humans and animals (13, 14). However, in recent years, multiple lines of evidence came from several well-designed studies mainly based on molecular Koch's postulates, which demonstrated the critical role of *CPA* in the pathogenesis of bovine necro-hemorrhagic enteritis (15–17). *C. perfringens* toxinotypes D isolates tend to produce alpha- and epsilon-toxin, which are encoded by plc and ε*-toxin* gene leading to the development of enterotoxemia (“pulpy kidney”) in sheep and goats (18–20).

Optimization is a method for response surface methodology (RSM) that focuses on the set of independent variables that serve as a mediator for discovering optimum conditions for the best possible response. This method is applied to gain maximum benefits from a particular process thus improving the performance of a system. This method could serve as a predictive study for determining the incidence of prevalent toxinotypes produced by *C. perfringens* (21). Many types of vaccines, medicines, and other biological agents have been tested for optimization to make the best combination that could be beneficial to every consumer (22). It consists of a polynomial model that is validated by variables for a particular response. It has a vast application in the field of microbiology and veterinary sciences. In this study, the toxinotypes of *C. perfringens* were subjected to an optimization process by Box-Behnken design (BBD) and central composite design (CCD) to determine the abundance of clostridial toxins (*type A* and *type D*) in *B. bubalis* and *B. indicus*.

*C. perfringens type D* (*epsilon toxin*) is responsible for enterotoxemia in sheep, goats, and cattle (19). ε*-toxin* is synthesized as a single-chain inactive prototoxin of 32–33 kDa in the gastrointestinal lumen, which is subsequently converted into an active toxin (29 kDa) by serine-type proteolytic enzymes (notably α*-chymotrypsin, trypsin*, and λ*-protease*). Once activated in the intestinal tract, ε*-toxin* increases intestinal vascular permeability, which in turn facilitates the entry of the toxin into the bloodstream. This event provides an opportunity for activated ε*-toxin* to rapidly damage different organs, including the brain, kidney, and lungs, through affecting vascular permeability and direct action on tissues (23). ε*-toxin* gene is encoded on the plasmids and is often regarded as the third-most potent toxin produced by Clostridial species after tetanus and botulinum toxins (24), with the epsilon toxin gene ε*-toxin* typically encoded on plasmids. Pathotyping is a traditional procedure of classification of *C. perfringens* (25). Over the past few years, advanced techniques like pulsed-field gel electrophoresis (PFGE) and multilocus sequence typing (MLST) have been introduced, with the capacity to ascertain the emergence and spread of infectious agents and explicate the immanent epidemiology of bacterial species (26, 27). Amid several accessible molecular typing tools, PFGE is still one of the most significant third-generation technique to decrypt the genetic diversity principally all of the bacterial species and also concede as gold standard due to brilliant discriminatory power and reliability of procedures upon developing macro-restriction pattern of the bacterial genome (28, 29).

The study was designed to assess the abundance of major toxin-producing genes related to *C. perfringens* using PFGE and check the interrelationship of pulsotypes to determine the genetic diversity to incide disease in buffalo and cattle of Pakistani origin.

## Materials and Methods

### Site Selection

**Figure 1:**
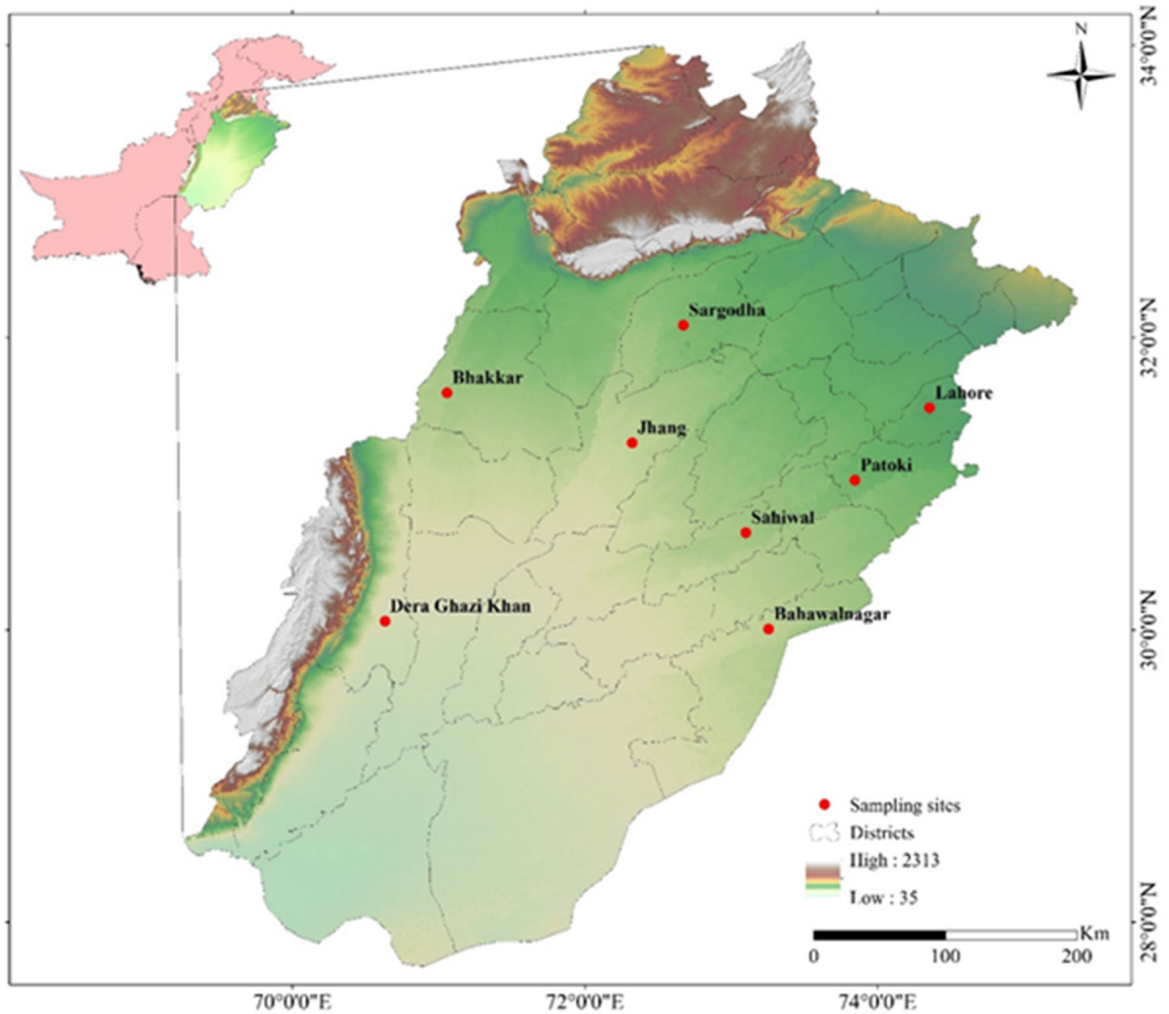
Map showing sampling sites (Buffalo, Cattle) of different cities of Punjab province, Pakistan generated by ArcGIS 10.2.2 software (ESRI, Redlands, CA, USA) by using Shuttle Radar Topography Mission (SRTM) Digital Elevation Model.

The sites for sampling were divided into four sampling zones, namely, Zone-I (Lahore, Pattoki) Zone-II (Sargodha, Jhang), Zone-III (Sahiwal, Bahawalnagar), and Zone-IV (Bakkhar, Dera-Ghazi Khan). These zones are considered rich regarding buffalo and cattle population (Figure 1).

### Sample Collection

A total of 160 animals comprising of 80 buffalo and 80 cattle were used in the study. Out of total 160 animals, 18 buffalo, and 15 cattle were found infected with *C. perfringens*. Intestinal contents (small intestine) of necropsied buffalo (*n* = 18) and cattle (*n* = 15) with a history of the intestinal problem were collected in sampling bags and transported in an ice box with a maintained temperature to the laboratory for further processing. Gross pathology is characterized by segmental to diffuse, hemorrhagic, or necrotizing inflammation of small intestinal mucosa.

### Isolation of Bacterial Strains

Fecal swabs infused with intestinal contents were inoculated into 5 ml thioglycollate (FTA) broth and incubated at 37°C (Don Whitely DG-250 anaerobic workstation, UK) for 24 h. Subsequently, 100 μl of pre-enriched FTA broth was spread on tryptose sulphite cycloserine agar base enriched with 7% egg yolk and supplemented with D-cycloserine (Solarbio, Beijing, China). Petri plates containing multiple black colonies harboring lecithinase positive reaction were selected and cultured. For identification and purity of *C. perfringens*, streaking was done on Columbia blood agar [Huan Kai Microbial (HKM) Sci & Tech, Guangzhou, China] containing 5% defibrinated sheep blood and evaluated for typical double zone hemolysis associated with *C. perfringens* isolates. Moreover, Gram staining and biochemical tests, i.e., glucose, maltose, H_2_S reduction test, nitrate reduction test, gelatin liquefaction, and saccharose test (Hangwei, Microbiological Co. Ltd., Hangzhou, China) were performed. Isolates were preserved in 50% glycerol at −80°C for further use.

### Extraction of Bacterial DNA

Genomic DNA of *C. perfringens* was extracted from overnight FTA broth culture inoculated with a single colony plate by Ultraclean Microbial DNA Isolation Kit (MoBio, Germantown, Maryland, USA) rendering to the manufacturer's directives with minor modification to attain high concentration. In brief, the bacterial suspension and lysis buffer mixed in addition of 20 μl of 20 mg/ml proteinase-K (Fisher Scientific, USA). Quality and quantity was determined by NanoDrop™ 2000 (Thermo Scientific Inc. Waltham, MA, USA). Protein contamination and the recommended purity level was estimated at the A260/A280 ratio of 1.8. The DNA was stored at −20°C for further genotyping analysis.

### 16S rRNA Gene Sequence Analysis

The 16S rRNA gene was amplified with primer from the genomic DNA of each strain using 16S rRNA gene species-specific primer (3). The sequences of the 16S rRNA gene region were determined by Sanger sequencing (Tsingke Biotechnology Company, Xian, China). The species were recognized by nucleotide alignments with the sequences of species banked in the GenBank NCBI, USA.The primers and conditions of amplification and sequencing of 16S rRNA genes references provided by Meer and Songer (30) and Kikuchi et al. (31), respectively. Primer sequence for genes regarding 16SrRNA (Table 1).

**Table 1:** Primers used for sequencing of genes.

**Gene**	**Primer sequence**	**References**
*plc*	GCTAATGTTACTGCCGTTGACC CCTCTGATACATCGTGTAAG	(30)
*etx*	CCACTTACTTGTCCTACTAAC GCGGTGATATCCATCTATTC	
16S rRNA gene	AGAGTTTGATCCTGGCTCAG TACGGYTACCTTGTTACGACTT	(31)

### Assessment of Toxin Production by *C. perfringens* Isolates

Primers corresponding to the α *toxin* (*CPA*), β *toxin* (*cpb*), or ε *toxin* (ε*-toxin*) gene were used as described by Svensson et al. (4). Genes encoding toxin proteins, including *cpb2*, were detected by PCR (5–7). American Type Culture Collection ATCC-3624 (*toxin type A*, α*-toxin positive*) and China Institute of Veterinary Drug Control, Beijing, China, including CVCC-54 (*toxin type B*, α*-*, β*-, and* ε*-toxin positive*), CVCC-61 (*toxin type C*, α*-, and* β *toxin positive*), and CVCC-81 (*toxin type D*, α*- and* ε *toxin positive*) were used as reference strains for toxinotyping (*A, B, C, and D, respectively*), and as positive controls for *cpb2* and *cpe*. Amplification was performed in a 25 μl reaction mixture containing 50 ng template DNA, PCR Premix Taq (Ex Taq V.2.0 plus Dye Takara, Japan), and 0.5 μM of each primer on a thermocycler (Takara, Japan); the total reaction volume was adjusted with the addition of RNase free water. The reaction conditions were as follows: initial denaturation at 96°C for 5 min; 35 cycles of denaturation at 96°C for 1 min., annealing at 56°C for 1 min and elongation at 72°C for 1 min; and a final extension at 72°C for 10 min.

For ε*-toxin*, the assay conditions were modified as follows: initial denaturation at 95°C for 3 min; 35 cycles of denaturation at 95°C for 40 s, annealing at 57°C for 30 s, and elongation at 72°C for 30 s; and a final extension at 72°C for 5 min. The amplified products were analyzed on a 1.2% agarose gel stained with ethidium bromide (10 mg/ml) by (GenStar, Beijing, China) PCR-amplified products on the gels were extracted and purified by E.Z.N.A® Gel Extraction Kit (Omega Bio-Tek, USA), and sequenced (Tsingke Biotechnology Company Xian, China) to ensure the identity with reference sequences.

### Genotyping of *C. perfringens* by PFGE

PFGE was performed according to the method described by Gholamiandekhordi et al. (12) with slight modifications (8). Inserts of genomic DNA were digested with 37°C for 2.5 h with 20 U of *SmaI* enzyme. Electrophoresis was performed in a CHEF-DRIII apparatus (Bio-Rad, USA). DNA fingerprints were compared by the BioNumerics 7.6, Austin, TX, USA, using a cut-off value with 90% similarity based on Dice coefficient identify PFGE genotypes. Plug preparation, restriction digestion, and electrophoresis conditions were essentially performed for *C. perfringens* according to the previously described protocol by Lindström et al. (6). Cells were suspended in suspension buffer [75 mM NaCl and 25 mM EDTA, (pH 8.0)] to an estimated absorbance of 1.3 at 610 nm. Bacterial cells were embedded in 1.2% chromosomal-grade Pulse Field Certified Agarose (Bio-Rad, USA) by mixing equal volumes (0.5 ml) of the cell suspension and melted agarose equilibrated to 55–65°C. In some preparations, bacterial cells were pretreated with formalin to reduce the interference by endogenous *DNase* activity before they were mixed with agarose.

Plugs were solidified at 4°C in 1.5 mm thick molds (Bio-Rad Laboratories, Hercules, CA, USA). The agarose-embedded cells were lysed by incubation of the plugs overnight at 55°C with gentle shaking in lysis buffer comprising 50 mM Tris-HCl [pH 8.0], 50 mM EDTA [pH 8.0], 1% N-lauryl sarcosine, and *proteinase K* (1 mg/ml Merck, Germany). The plugs were washed three times at 50°C with vigorous shaking for 15 min each in sterile reagent-grade water followed by three times of washing with TE buffer comprising of 10 mM Tris, 1 mM EDTA (pH 8.0) Tris EDTA for 15 min each time. Plugs were stored in TE buffer at 4°C in refrigerator for further experimental usage. These plugs were cut into 3–4 mm slices and equilibrated with appropriate restriction buffer containing (20 mg/ml) bovine serum albumin (BSA, Amresco, Solon, Ohio, USA). The *SmaI* (New Bio, England) restriction enzyme was used at appropriate conditions recommended by the manufacturer. Restriction fragments were separated by electrophoresis through a 1% Pulse Field Certified Agarose (Bio-Rad, USA) solution of Tris-borate and EDTA (TBE buffer, Solarbio, Beijing, China). TBE buffer containing 200 μM thiourea (Sigma-Aldrich, St. Louis, MO, United States) at 14°C in a contour-clamped homogeneous electric field MAPPER XA PFGE (Bio-Rad, USA) apparatus was used to increase the band quality and resolution. The run time was 18 h with a voltage of 6 V/cm and a linearly ramped pulse time of 0.5–38 s. The restricted fragments were separated in a 1% agarose gel in 0.5 × TBE buffer by using a CHEF-DR III system (Bio-Rad, USA). Following the electrophoresis, the gel was stained in aqueous ethidium bromide (10 mg/ml) by (GenStar, Beijing, China) followed by a destained step in water for 20 min, and the image was captured by a ChemiDoc CRX+ Image analyzer (Bio-Rad USA) as TIFF files.

### PFGE Agarose and DNA Migration

Based on equations and reprise DNA model migration in traditional electrophoresis, the linear DNA molecular i (Di) migration in a counter-clamed homogeneous electric field (CHEF) dynamic regulation (DR-III) gels are described by Lindström et al. (6), Roeder et al. (10), Canard et al. (11), Lindström et al. (6), Roeder et al. (10), and Canard and Cole (11).

### Computational Analysis of PFGE Patterns

An analysis obtained from the restrictive *SmaI* endonuclease was carried out with PFGE standards and analyzed using BioNumerics version 7.6 (Applied Math's, Inc., Austin, TX, USA). The assessment was based on a band for each *type A* and the similarity analysis was performed using the Dice coefficients (SD) with a custom tolerance of 1.5%. The type of dendrogram was made by unweighted pair group impressions formed by the unweighted pair group method with arithmetic mean (UPGMA). The discriminatory power was calculated by the Simpson diversity index (S_D_) (32).

### Statistical Analysis

Analysis of data was performed by one-way analysis of variance (ANOVA) along with the means of treatments were compared using Tukey's Honestly Significant Difference (HSD) test using SPSS [Statistical Package for Social Sciences (SPSS) version 26.0 Armonk, NY, USA]. Correlation, regression, and cluster analysis using heatmap were performed using R Studio suite 1.3.1093. Optimization of the parameters was done using RSM *via* CCD and BBD using the Design-Expert software (Design Expert® version 12.0, Stat-Ease Inc. Suite 6400, Minneapolis, MN55413, USA).

## Results

### Distribution of *C. perfringens* From Four Sampling Zones of Punjab Province in Pakistan

A total of 160 animals (buffalo = 80 and cattle = 80) were examined for *C. perfringens* infection. Of the total animals, only positive tested samples were isolated from both buffalo and cattle. The zonal distribution of collected samples is stated in Table 2. On processing the collected samples, after careful isolation and identification (classical method, i.e., culturing, biochemical tests), we performed PCR of toxin-encoded genes to confirm their serotypes (8). Furthermore, PFGE performed with *SmaI* that gives different fingerprint patterns leads to the development of 26 pulsotypes (PFGE patterns) which indicates genetic diversity among study samples.

**Table 2:** Distribution of *C. perfringens* in buffalo and cattle from four sampling zones of Punjab province in Pakistan.

**Zones of sampling**	**Buffalo (** ***B. bubalis*** **)**		**Cattle (** ***B. indicus*** **)**	
	**Number**	**Percentage %**	**Number**	**Percentage %**
Zone-I (Lahore, Pattoki)	5	27.78	4	26.67
Zone-II (Sargodha, Jhang)	4	22.22	2	13.33
Zone-III (Sahiwal, Bahawalnagar)	3	16.67	4	26.67
Zone-IV (Bhakkar, Dera-Ghazi Khan)	6	33.33	5	33.33
Total	18		15

### Isolate Isolation and Identification

Culturing, biochemical testing, i.e., glucose (+), maltose (+), H_2_S reduction test (–), nitrate reduction test (+), gelatin liquefaction (+), and saccharose test (+), amplification (PCR), and subsequent sequencing of the 16S rRNA gene confirmed that all 33 isolates were of *C. perfringens*. The representative sequences can be accessed at NCBI under accession number MT158886-MT158897.

### Cluster Analysis of *C. perfringens* Based Toxinotypes in Buffalo and Cattle From Four Zones in Punjab Province

**Figure 2:**
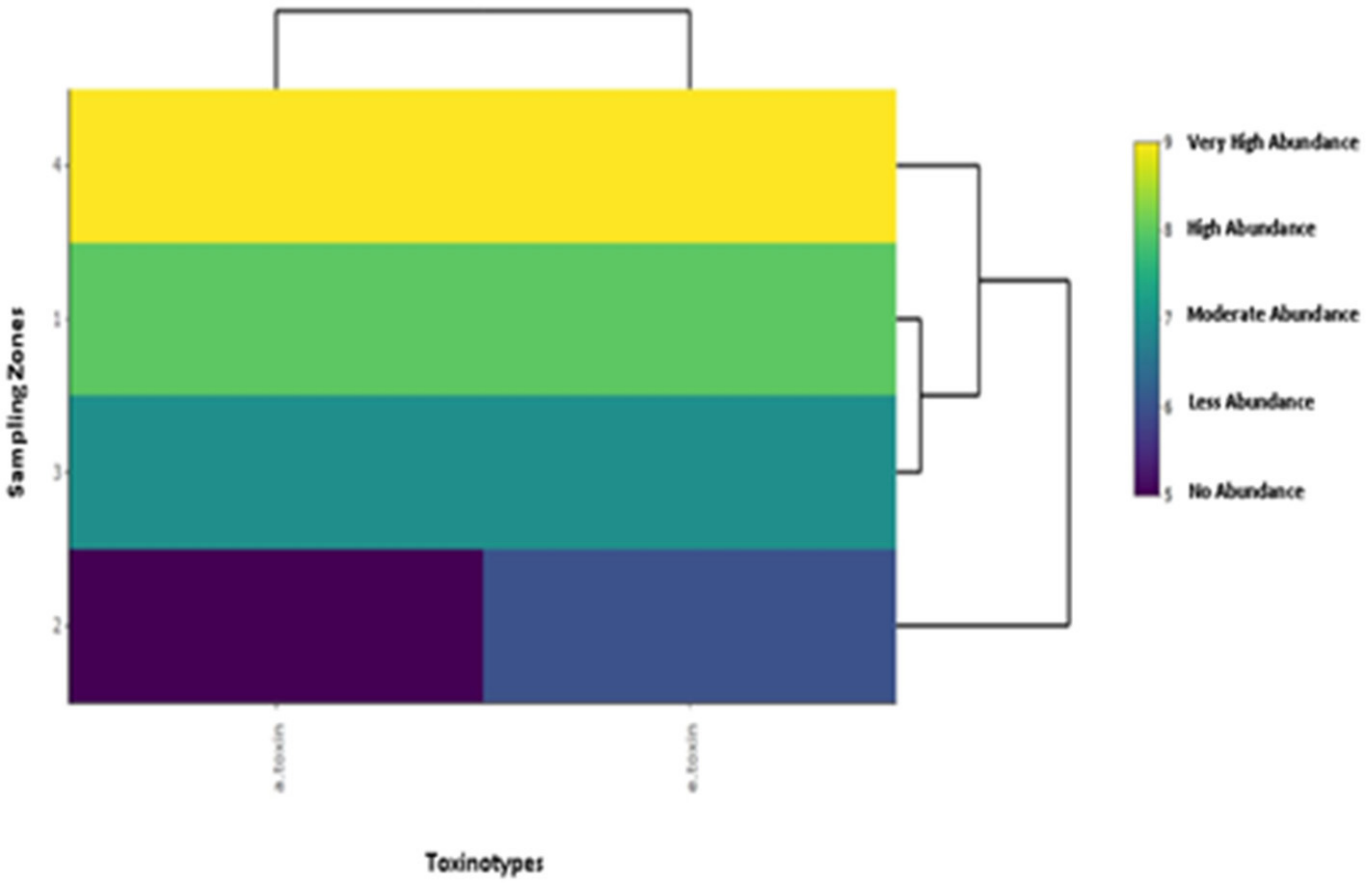
Illustration of cluster analysis by heatmap of toxinotypes in *C. perfringens* isolated from buffalo and cattle in four different zones of Punjab province in Pakistan. The color scheme represents the abundance of toxinotypes in buffalo and cattle belonging to four sampling zones.

For cluster analysis of the toxinotypes in *C. perfringens*, a heatmap was used. In the case of α*-toxin* and ε*-toxin* of *C. perfringens*, the heatmap depicts that zone-IV is abundant in both toxinotypes, while Zone-II is less abundant. α*-toxin* was mostly observed in cattle and buffalo belonging to zone-IV, while ε*-toxin* was also found maximum in cattle and buffalo belonging to zone-IV. This illustration depicts that buffalo and cattle of Zone-IV are more vulnerable to *C. perfringens* (Figure 2).

### Optimization of *C. perfringens* Toxinotypes (*α-Toxin and ε-Toxin*) in Sampled Zones of Punjab Province by Response Surface Methodology

For the toxinotypes, the abundance in buffalo and cattle was determined based on RSM *via* CCD. The coding of variables is shown in Table 3. The data were applied to the following equation:


(1) $$\begin{array}{l} \text{Y}=\beta_{o}+\overset{2}{\underset{i=1}{\sum }}\beta_{ii}F_{i}+\overset{2}{\underset{i=1}{\sum }}\beta_{ii}F_{i}^{2}+\sum \overset{2}{\underset{i<j=1}{\sum }}\beta_{ij}F_{i}F_{j}+\varepsilon \end{array}$$

where “Y” is the abundance of *C. perfringens* toxinotypes., “β_0_” is the intercept constant, “βi,” “βii,” “βij” are the regression coefficients of “F_1_,” “F_2_,” “F_i_,” “F_j_” are coded values of independent variables, which are α*-toxin* and ε*-toxin* (Table 3), respectively, while “ε” is the error term.

**Table 3:** The design approach for determining the optimization of *C. perfringens* toxinotypes in central–composite design (CCD).

**Factors**	**Coded symbols**	**Levels**	
		**−1**	**+1**
α-toxin (plc)	A	5	9
ε-toxin (etx)	B	6	9

**Figure 3:**
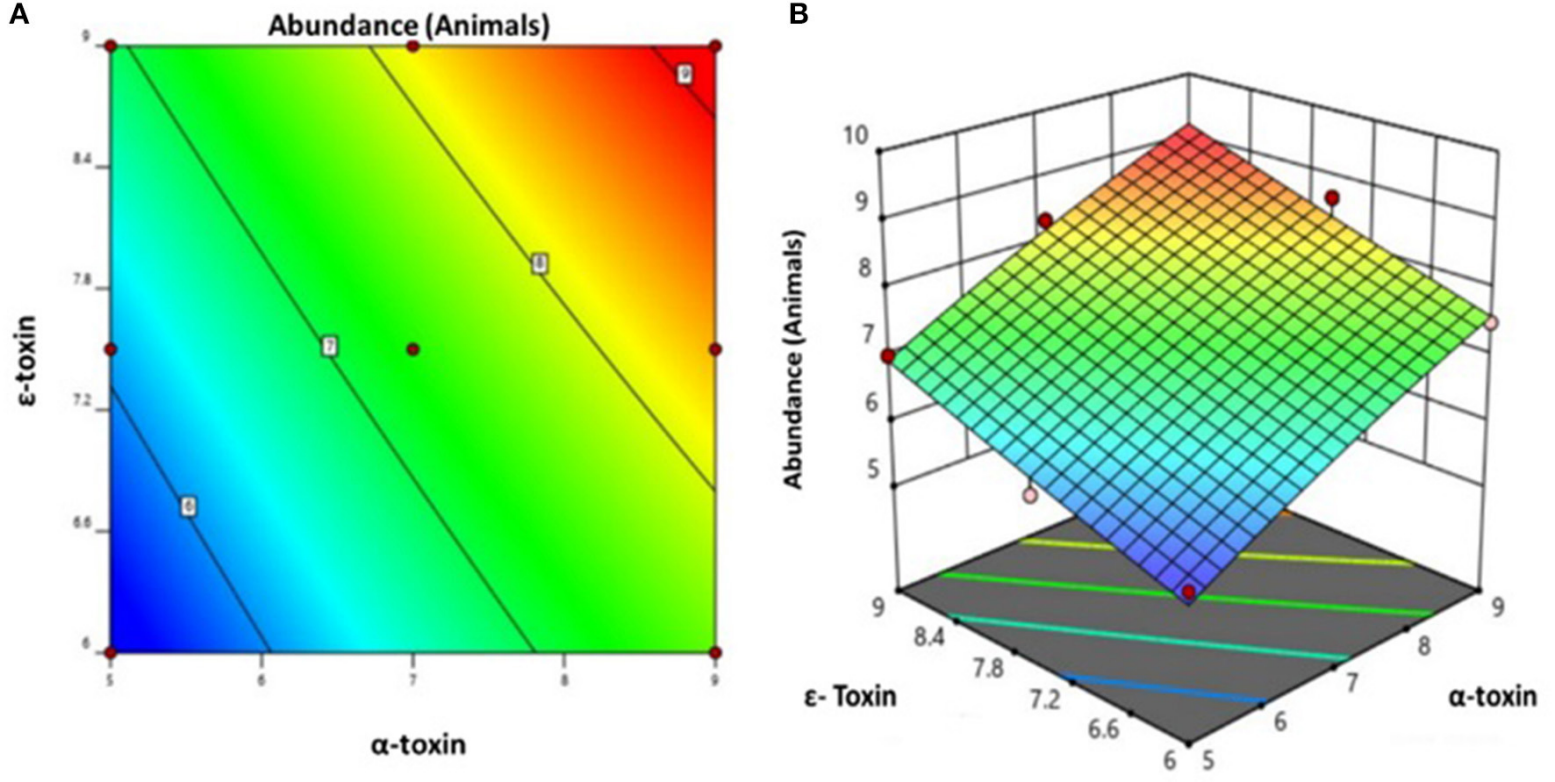
Prevalence of *C. perfringens* toxinotypes in four different zones of Punjab province in Pakistan **(A)** contour plot **(B)** surface plot.

By applying ANOVA (Table 4) for the model, it was found to be significant (*P* < 0.05). It indicated that this model was adequate and reproducible. The abundance of *C. perfringens* predicted by the regression equation was close to the observed ones (*R*^2^ = 0.98). Based on the ANOVA, two independent variables, namely, α*-toxin* and ε*-toxin* had a significant linear effect and quadratic effect on abundance. The above parameters for abundance of *C. perfringens* were evaluated concluding an optimized value could be achieved at α*-toxin* level of 7 and ε*-toxin* level of 5. In Figure 3, the zones of optimization are shown in the contour and surface plots to illustrate the effects of independent variables (factors), i.e., α*-toxin* and ε*-toxin* on the dependent variable (response), i.e., abundance in cow and buffalo. The interactive effect of these toxinotypes was found to be nonsignificant at *P* < 0.05. Both α*-toxin* and ε*-toxin* have their own impact. Runs 1, 5, and 8 of table display low abundance, while other runs illustrate high abundance of α*-toxin* and ε*-toxin*, respectively (Table 5).

**Table 4:** Analysis of variance for prevalence of *C. perfringens* toxinotypes in four different zones of Punjab province in Pakistan.

**Source**	**Sum of squares**	**df**	**Mean square**	***F*** **-Value**	***p*** **--value**
Model	12.23	5	2.45	26.83	0.0108
A-α-toxin	7.77	1	7.77	85.27	0.0027
B-ε-toxin	4.12	1	4.12	45.19	0.0067
A × B	0.0000	1	0.0000	0.0000	1.0000
A^2^	0.0200	1	0.0200	0.2198	0.0167
B^2^	0.0017	1	0.0017	0.0187	0.0399
Residual	0.2734	3	0.0911		
Lack of fit	0.0034	2	0.0017	6.29 × 10^−3^	0.912
Pure error	0.27	1	0.27		
Total	12.5034	8			

**Table 5:** Actual and predicted values for the abundance of *C. perfringens* toxinotypes in four different zones of Punjab province in Pakistan.

**Runs**	**A: α-toxin**	**B: ε-toxin**	**Abundance (animals)**	
			**Actual values**	**Predicted values**
1	7	5	6	5
2	9	8	9	8
3	5	9	7	6
4	7	9	8	7
5	5	6	6	5
6	7	8	7	6
7	9	6	8	7
8	5	8	6	5
9	9	9	9	8

Based on the ANOVA, the following regression equation was obtained for the optimization of *C. perfringens* toxinotypes:


(2) $$\begin{array}{l} \text{Y = }4.75\;+\;0.75\text{A }+\;0.125\text{B }+\;0.645\text{C }+\;0.771\text{D }+\;0.125\text{AB}\\ \;-\;0.313\text{AC}-0.0625\text{AD}+0.125\text{BC}+0.125\text{BD}-0.25\text{CD}\\ \begin{array}{cc} \;+ & 0.0624{\text{A}}^{2}+0.0624{\text{B}}^{2}+0.156{\text{C}}^{2}-0.156{\text{D}}^{2} \end{array} \end{array}$$

### Optimization of Sampling Zones Against the *C. perfringens* Toxins by Response Surface Methodology

For all the zones of sampling, the abundance was assessed based on RSM *via* Box Behnken design (BBD). The coding of variables is shown in Table 6. The data were applied to the following equation:


(3) $$\begin{array}{r} Y=\beta_{o}+\overset{3}{\underset{i=1}{\sum }}\beta_{ii}F_{i}+\overset{3}{\underset{i=1}{\sum }}\beta_{ii}F_{i}^{2}+\sum \overset{3}{\underset{i<j=1}{\sum }}\beta_{ij}F_{i}F_{j}+\varepsilon \end{array}$$

where “Y” is the abundance in buffalo and cattle, “β_0_” is the intercept constant, “βi,” “βii,” “βij” are the regression coefficients of “F_1_,” “F_2_,” “F_3_,” “F_i_,” “F_j_” are coded values of independent variables which are four sampling zones in Punjab Province, respectively, while “ε” is the error term.

**Table 6:** The design approach for determining the optimization of four sampling zones in the abundance of *C. perfringens* in Box–Behnken Design (BBD).

**Factors**	**Coded symbols**	**Levels**	
		**−1**	**+1**
Zone-I	A	1	8
Zone-II	B	1	6
Zone-III	C	1	7
Zone-IV	D	1	9

**Figure 4:**
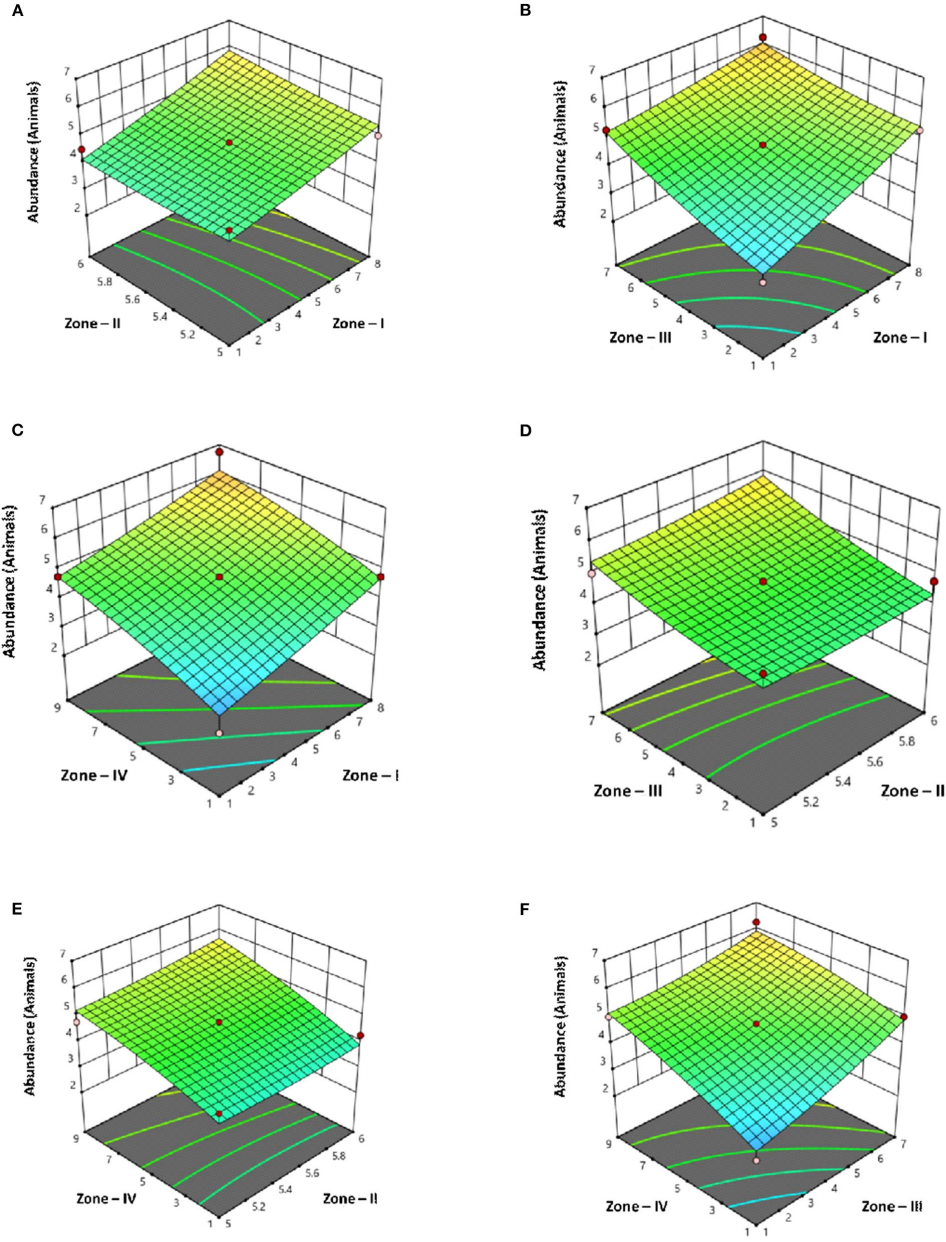
Surface plots of *C. perfringens* toxins in four different zones with Punjab province in Pakistan with their interaction **(A)** Zone–I × Zone–II **(B)** Zone–I × Zone–III **(C)** Zone–I × Zone–IV **(D)** Zone–II × Zone–III **(E)** Zone–II × Zone–IV **(F)** Zone–III × Zone–IV.

By applying ANOVA (Table 7) for the model, it was found to be significant (*P* < 0.05). It indicated that this model was adequate and reproducible. The abundance of *C. perfringens* toxins predicted by the regression equation was close to the observed ones (*R*^2^ = 0.99). Based on the ANOVA, four independent variables, namely, zone-I, zone-II, zone-III, and zone-IV had a significant linear effect and quadratic effect on the abundance of toxins in animals. The above parameters for abundance were evaluated concluding an optimized value could be achieved at 1 unit of zone-I, 6 units of zone-II, 1 unit of zone-III, and 5 units of zone-IV. In Figure 4, the zones of optimization are shown in the surface plots to illustrate the effects of independent variables (factors), i.e., sampling zones on the dependent variable (response), i.e., abundance in animals. The interactive effect of all zones was found to be positive and significant regarding the abundance of toxins in cows and buffalo and the significance value was found to be <5%. All these factors show relatively significant abundance in both buffalo and cattle. In runs 14, 18, and 25, the animals showed less abundance of toxinotypes based on four zones of sampling (Table 8).

**Table 7:** Analysis of variance for the prevalence of *C. perfringens* toxinotypes in four different zones of Punjab province in Pakistan.

**Source**	**Sum of squares**	**df**	**Mean square**	***F*** **--Value**	***p*** **--value**
Model	20.34	14	1.45	4.94	0.0077
A-Zone-I	6.75	1	6.75	22.94	0.0007
B-Zone-II	0.1875	1	0.1875	0.6372	0.0433
C-Zone-III	5.01	1	5.01	17.01	0.0021
D-Zone-IV	7.13	1	7.13	24.23	0.0006
A × B	0.0625	1	0.0625	0.2124	0.0448
A × C	0.3906	1	0.3906	1.33	0.0261
A × D	0.0156	1	0.0156	0.0531	0.0224
B × C	0.0625	1	0.0625	0.2124	0.0148
B × D	0.0625	1	0.0625	0.2124	0.0148
C × D	0.2500	1	0.2500	0.8496	0.0184
A^2^	0.0110	1	0.0110	0.0375	0.0485
B^2^	0.0110	1	0.0110	0.0375	0.0485
C^2^	0.0689	1	0.0689	0.2343	0.0388
D^2^	0.0689	1	0.0689	0.2343	0.0388
Residual	2.94	10	0.2943		
Lack of fit	1.90	8	0.2375	0.45	0.792
Pure error	1.04	2	0.52		
Total	23.29	24			

**Table 8:** Actual and predicted values for *C. perfringens* toxins in four different zones of Punjab province in Pakistan.

**Runs**	**A: Zone-I**	**B: Zone-II**	**C: Zone-III**	**D: Zone-IV**	**Abundance (animals)**	
					**Actual value**	**Predicted value**
1	1	6	4	5	5	4
2	5	5	4	1	4	4
3	8	6	7	5	6	6
4	8	6	4	9	7	6
5	8	6	1	5	5	5
6	5	6	4	1	4	4
7	1	6	7	5	5	5
8	5	5	7	5	5	5
9	5	6	1	5	5	4
10	8	5	4	5	5	5
11	1	6	4	9	5	5
12	5	6	4	9	5	6
13	5	6	1	9	5	5
14	1	6	1	5	3	3
15	5	6	7	1	5	5
16	5	6	7	9	6	6
17	5	6	7	5	6	6
18	1	6	4	1	3	3
19	1	5	4	5	5	4
20	5	5	4	9	5	5
21	5	5	1	5	5	4
22	5	6	4	5	5	5
23	8	6	4	5	6	6
24	8	6	4	1	5	5
25	5	6	1	1	3	3

**Figure 5:**
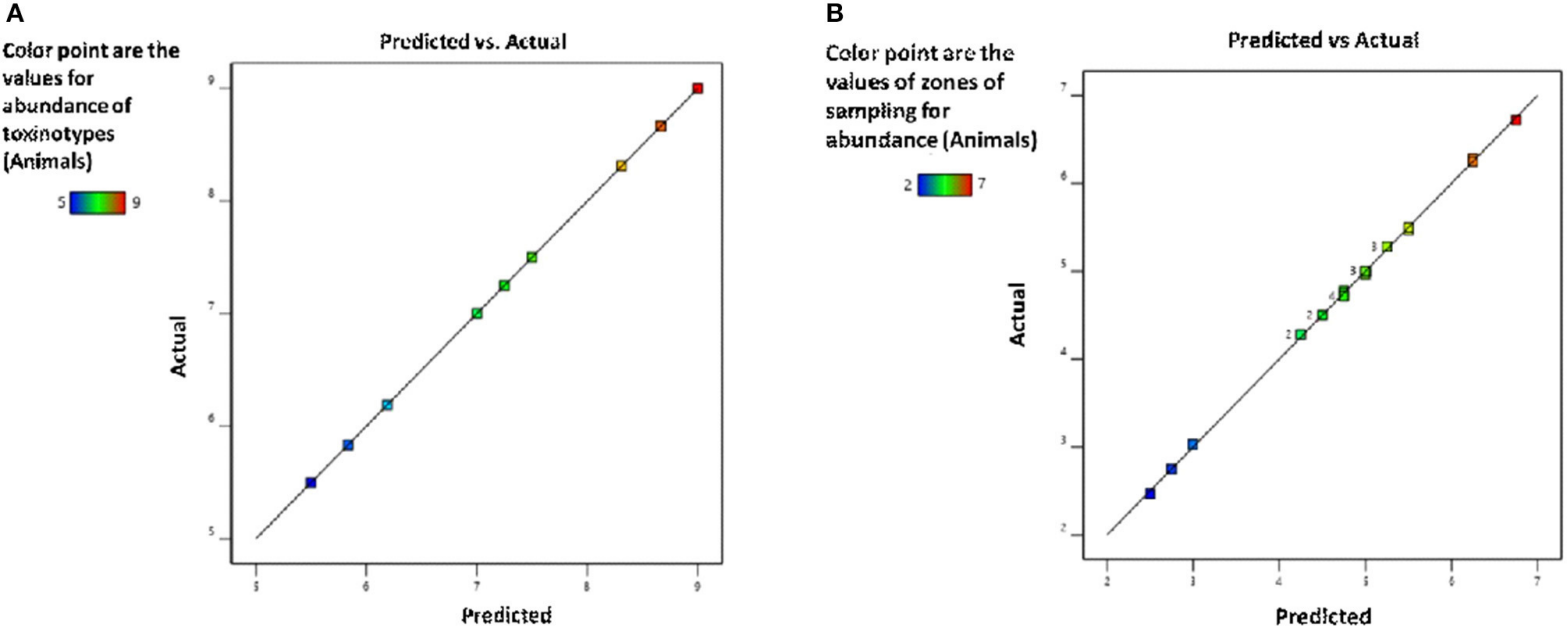
Validation of the model for **(A)** toxinotypes and **(B)** zones of sampling for the assessment of *C. perfringens* abundance in the buffalo and cattle population of Punjab province, Pakistan.

Figure 5 and Table 9 illustrate the summary statistics of the model applied to the variables, which clarifies the significance of both RSM-based designs.

**Table 9:** Summary statistics for responses used in CCD and BBD.

**Source**	***R^**2**^***	**Adjusted *R^**2**^***	**Predicted *R^**2**^***	**Remarks**
**Model summary statistics for toxinotypes**				
Linear	0.9760	0.9417	0.7736	
Quadratic	0.9781	0.9681	0.9464	Suggested
Cubic	0.9546	0.9231	0.8564	Aliased
**Model summary statistics for sampling zones**				
Linear	0.8736	0.6967	0.6324	
Quadratic	0.9993	0.9919	0.9837	Suggested
Cubic	0.9181	0.7829	0.7127	Aliased

Based on the ANOVA, following regression equation was obtained for the optimization of sampling zones:


(4) $$\begin{array}{l} \begin{array}{ccc} \text{Y} & = & 7.33+1.13\text{A}+0.805\text{B}+0.00\text{AB}-0.103{\text{A}}^{2} \end{array}\\ \;\begin{array}{cc} + & 0.023B^{2} \end{array} \end{array}$$

### Toxinotyping and Confirmation of Genes Encoding Toxin Proteins by PCR

In the current study, isolates were divided into four sampling zones, namely, Zone-I, Zone-II, Zone-III, and Zone-IV. These zones are considered rich regarding buffalo and cattle population. The toxinotypes were confirmed by toxin-specific PCR and sequencing. The results revealed that the 33 isolated *C. perfringens* strains were either *type A* or *type D*. Major proportion (55%, 28/33) belonged to *type D* (*plc*+, ε*-toxin*+), while only 15% (5/33) of samples were identified as *type A* (*plc*+).

### Pulsed Field Gel Electrophoresis

**Figure 6:**
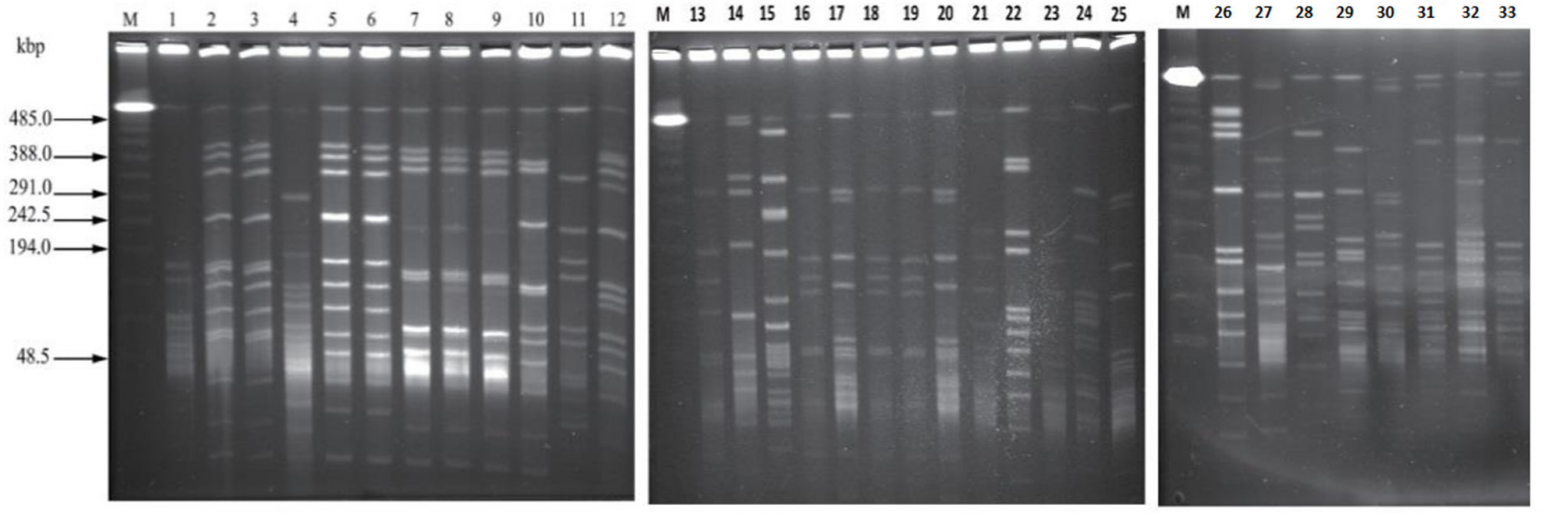
The pattern obtained by PFGE SmaI, Marker = (PFGE Lambda Marker New England Bio, UK) lanes: 1–33 strains of *C. perfringens* of bovine origin showing different DNA macrorestriction pattern.

Analysis of 33 *C. perfringens* strains provided reasonable pattern that led to formation of 26 pulsotypes showed higher discriminatory power of *SmaI* digestion activity and proved to be capable to *C. perfringens type A* strains to achieve the satisfactory type ability. Restriction enzyme profiles of the isolates' genomic DNA obtained by using *SmaI* showed 9–13 fragments ranging in size from ~48 to 485 kb (Figure 6).

**Figure 7:**
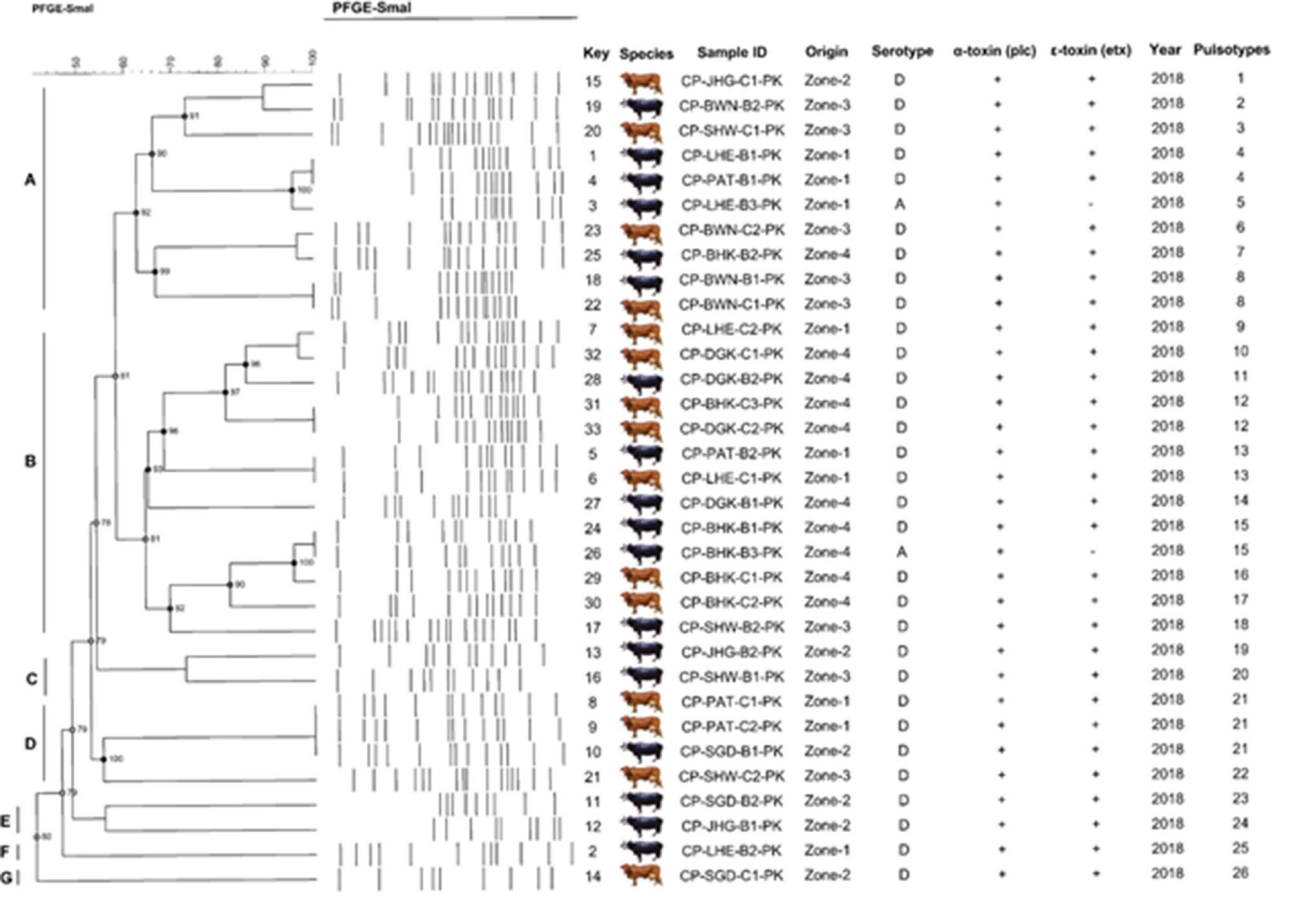
Schematic representation (genetic profile, key species, isolate, serotype, toxinotypes, and pulsotypes) of 33 *C. perfringens* isolates obtained from buffalo and cattle in Pakistan. The macro-restriction pattern was conducted with SmaI. Dendogram obtained using BioNumerics 7.6 software and unweighted pair group method with Arithmetic mean. Letters **(A–G)** represents the *C. perfringens* toxinotypes prevalent in bovines.

According to the criteria of Layana et al. (18) and Tenover et al. (33), none of the isolates can be considered genetically indistinguishable. Two isolates were closely related, with 2- to 3-band differences. Dendrogram constructed, considering the adopted UPGMA parameters, grouped the isolates into seven different clusters (A to G) with the main clusters having similarities ranging from 80 to 95% (BioNumerics 7.6, Austin, TX, USA) and generated 26 pulsotypes based on the band (line) pattern (Figure 7). We collected the field samples and performed careful isolation and characterization *via* PCR and found *type A* (*plc*) and *type D* (*plc* + *etx*) on the basis of toxin-encoded gene amplification. On processing of collected samples, after careful isolation and identification (classical method, i.e., culturing, biochemical tests), we performed PCR of toxin-encoded genes to confirm their serotypes (8). Furthermore, PFGE performed with *SmaI* that gives different fingerprint patterns leads to development of 26 pulsotypes (PFGE patterns) that indicates genetic diversity among study samples.

Cluster A was found to be highly diverse among the others, as it consisted of the samples from all the sampling zones included in the study. Cluster D and F consisted of only buffalo isolates, while the rest of the clusters included isolates from both buffalo and cattle. All isolates were found to be α*-toxin* and ε*-toxin* positive, while only 5 were ε*-toxin* negative. Out of 5 ε*-toxin* negative samples, 4 were of buffalo origin, while only one was of cattle origin. The animals from Zone-I fashioned 4 similar genomic patterns of DNA macrorestriction patterns on parallel Zone-IV also produced 2 similar PFGE profiles. Our data provide information about running PFGE profile (pulsotypes) in dairy animals of Punjab Province, Pakistan.

### Simpson Index (S_D_) and Cophenetic Correlation (ρ)

A high S_D_ value was achieved with *SmaI* (S_D_ = 0.98) yielding 26 pulsotypes. The excellence of the cluster analysis was validated by the cophenetic correlation value (ρ) for the dendrogram. Each strain of DNA underwent duplication to verify the repeatability of the macrorestriction pattern of running DNA samples. PFGE profiles (pulsotypes) are given in the dendrogram provided by a cophenetic correlation coefficient (ρ) equal to 0.90. This explained the fitness and reliability of the clustering of original data presented by Muth and Morrill (20) and generally falls between 0.60 and 0.95 and values higher than 0.80 are regarded as satisfactory as reported by Hornitzky and Glastonbury (34) and Knapp et al. (35).

## Discussion

*C. perfringens* is an important part of intestinal microflora and certain strains are well-documented for triggering diseases in animals and humans varying from myonecrosis and food poisoning to enterotoxemia and enteritis (36, 37). The association of disease is augmented by ε*-toxin* in numerous animal species advocated with the clinical and pathological investigation (19, 38). Investigational trials related to enterotoxemia in cattle indicate that ε*-toxin* was the crucial factor in the pathogenesis of this disease in (bovine) dairy farm animals (19). Although *C. perfringens type D* secretes ε*-toxin* similarly, the synergistic role of additional *C. perfringens* toxins as α*-toxin* and other twenty minor toxins cannot be precluded (19, 36). ε*-toxin* is provocative among researchers, but all acquiesce that “pulpy kidney” is materialized in the kidney of ruminants with *type D* enterotoxemia (37, 39).

The toxin profiles and PFGE were used to characterize isolates obtained from buffalo and cattle feces. The PFGE profiles of all isolates differed by no more than two bands and the three patterns observed shared a 90% similarity. This is in contrast to the study by Akiba et al. (37). Statistics on enterotoxemia by *C. perfringens type D* in farm animals are limited, but a study conducted by Filho et al. (19) supports our result about a disease model in buffalo and cattle, which confirmed that the lethal ε*-toxin* is an elite cause of enterotoxemia in dairy farm animals. Our results impart support to that conjecture and anticipate sagacity related to genetic diversity.

The isolates that had PFGE pattern *type A* and *type D* were indisputably associated with the bovine origin and an identical pattern was obtained with a few isolates (40). In this study, the variation between the band patterns of PFGE types was reflected as adequate to discriminate between two different PFGE types (pulsotypes). The results gained by PFGE divulge a huge genetic diversity. No relationship was found between the isolated source and the positions in the dendrogram.

At present, the main advantage of PFGE application is to perform bacterial DNA fingerprinting that is preceding to differentiating bacterial subtypes. The bacterial genome analysis by PFGE provides remarkable information that helps to distinguish the clonality of the isolates in an outbreak to identify the origin, cause, and route along with broadcasting of the disease (12, 29, 41). Currently, PFGE is not only the point of focus for public health surveillance, disease cluster identification, and outbreak investigations but also considered the gold standard for bacterial subtyping. Moreover, PFGE gel upon completion of electrophoresis provides precise results in real-time. We assume that PFGE remains the key method for bacterial subtyping and performs molecular epidemiological studies in developed countries, although the switching to WGS will be a key obstacle due to the shortage of funds and the allocation of multidisciplinary qualified teams in several countries.

In this study, huge genetic diversity was possible because of the wide diversity of the isolates analyzed. PFGE clearly distinguished among disparate isolates of *C. perfringens* and established a clonal relationship between related strains. Although unrelated isolates impart 60 to 70% similarities, numerous fragments comigrated between isolates, similar to those reported by Canard and Cole (11), signifying that several sequences in the genome are conserved conversely; according to prior studies by Canard et al. (11) and Maslanka et al. (42), it was not astounding to observe different patterns between unrelated isolates. Computer-aided programs are used to obtain a more objective estimation of clonality between the strains. Visual assessment of subtype patterns leads to deduce the relatedness (band difference) when in fact the isolates were obtained in different cities of Pakistan. Khan et al. (43) and Nasir et al. (44) also confirmed the incidence of epsilon toxin causing eneterotoxemia in bovine species of Pakistan origin, which could be a major threat in the near future.

## Conclusion

The novelty of this study lies in the genomic diversity of *C. perfringens* by PFGE that would help us for better understanding related to epidemiology and disease control strategy. The establishment of deep knowledge at the genome level is required to figure out the host-pathogen interaction.

## Data Availability Statement

The datasets presented in this study can be found in online repositories. The names of the repository/repositories and accession number(s) can be found at: The representative sequences can be accessed at NCBI under accession number MT158886-MT158897.

## Ethics Statement

No animal studies are presented in this manuscript. However, the collection of intestinal contents from the necropsied cattle and buffaloes was done keeping the standards of animal welfare intact and strictly adhering to the international guidelines.

## Author Contributions

MZ, SK, and MH: conceptualization. MZ and MH: methodology, software, and writing—original draft. YL and JZ: validation, investigation, resources, supervision, project administration, and funding acquisition. SY: formal analysis. MZ, SK, MH, WA, MA, TM, MI, and MT: data curation. SY, YL, JZ, SK, WA, MA, TM, MI, and MT: writing—review and editing. SY, YL, and JZ: visualization. All authors contributed to the article and approved the submitted version.

## Funding

This study was supported by the Hebei Province Key R&D Program (Grant No. 21322913D), Gansu Province R&D Program (20YF3NA005), and the Project Funds (Nos. 2022YB009 and 2022YB010) supported by Hebei Normal University of Science and Technology.

## Conflict of Interest

The authors declare that the research was conducted in the absence of any commercial or financial relationships that could be construed as a potential conflict of interest.

## Publisher's Note

All claims expressed in this article are solely those of the authors and do not necessarily represent those of their affiliated organizations, or those of the publisher, the editors and the reviewers. Any product that may be evaluated in this article, or claim that may be made by its manufacturer, is not guaranteed or endorsed by the publisher.
